# How relaxed preferences facilitate the evolution of novel animal signals

**DOI:** 10.1093/evlett/qraf047

**Published:** 2025-12-08

**Authors:** Gabrielle T Welsh, James H Gallagher, Mary L Westwood, Vanessa Leon-Gamez, Lauren Bitner, Norman Lee, Robin M Tinghitella

**Affiliations:** Department of Biological Sciences, University of Denver, Denver, United States; Department of Evolution and Ecology, University of California Davis, Davis, United States; Department of Biological Sciences, University of Denver, Denver, United States; Department of Biological Sciences, University of Denver, Denver, United States; Department of Biology, St. Olaf College, Northfield, United States; Neuroscience Program, St. Olaf College, Northfield, United States; Department of Biology, St. Olaf College, Northfield, United States; Neuroscience Program, St. Olaf College, Northfield, United States; Department of Biological Sciences, University of Denver, Denver, United States

**Keywords:** sexual selection, mate choice, rapid evolution, communication, plasticity

## Abstract

The evolution of novel animal signals is critical to the generation of biodiversity. Here, we explore how new sexual signals become established. This process is challenging to explain because if receiver preferences are coupled with existing signals, then most receivers should discriminate against new signals. We investigated an underappreciated hypothesis: relaxed receiver preferences facilitate novel signal evolution by allowing new signals to establish a foothold. Further, we probed the mechanistic underpinnings of relaxed preferences by combining field-based and common garden approaches, allowing us to investigate evolution and plasticity as mechanisms. We capitalized on the Pacific field cricket, *Teleogryllus oceanicus*, a species that has recently evolved multiple novel acoustic signals (e.g., purring and rattling) in response to an eavesdropping parasitoid fly only found in the crickets’ introduced range in Hawaii. To test the hypothesis that selection associated with high search costs in introduced populations leads to relaxed mating preferences and determine whether such relaxation is plastic, we conducted sound preference (phonotaxis) trials with females from the cricket’s native range (Australia and French Polynesia, where the fly is absent) and its introduced range (Hawaii, where the fly is present). We presented females with novel songs plus the typical, ancestral song. Differences in phonotactic behavior between the lab and field settings would indicate plasticity in preferences. We found that Australian and French Polynesian females were quite plastic; they discriminated strongly against most songs in the field, but were much more phonotactic to rattling and the typical song in the lab. However, Hawaiian females exhibited little plasticity and were consistently highly responsive to the rattling and typical songs in the lab and field. This pattern points to a loss of ancestral plasticity in female preferences sometime after colonizing Hawaii, resulting in heightened responsiveness to all songs—allowing novel signals to establish.

## Introduction

The diversity of signals used in animal communication is vast and striking. Even among closely related species, sexual signals can be highly divergent, despite similarities in other fitness-related traits. Moreover, diversification, and even speciation, is believed to be tied to the evolution of sexual signals, making them particularly important to the origins and maintenance of biodiversity (Hund et al., 2020; Mendelson & Safran, 2021; Servedio & Boughman, 2017; West-Eberhard, 1983; Wilkins et al., 2013). Sexual selection contributes to speciation when both signals and receiver preferences become coupled within populations, but diverge between populations, generating reproductive isolation (Mendelson & Shaw, 2002; Panhuis et al., 2001). Thus, understanding when and how signal change occurs and why some new signals persist within populations reveals much about how animal communication contributes to biodiversity. We focus on the role of receiver preferences in the evolution of new and novel signals. We consider signals to be new if they are recently evolved and novel if they have a discontinuous origin and relatively abrupt deviation from the ancestral signal space (sensu Muller, 1990).

There are two general mechanisms by which new signals may become established: receiver-first mechanisms, where receivers have preferences for signal variants that do not yet exist, and signal-first mechanisms, where changes arise in the signaler first and receiver responses follow (Bradbury & Vehrencamp, 2011). Sensory bias, a receiver first mechanism, proposes that preferences for new signals may pre-date signal evolution because they evolved as by-products of receiver processing systems that were already tuned to stimuli important in other contexts such as foraging or predator avoidance (Endler & Basolo, 1998; Ryan et al., 1990). Similarly, the veiled preference hypothesis asserts that the discriminating sex evolves preferences (which do not have to be associated with a non-mating fitness advantage) that remain hidden until an associated novel trait evolves to take advantage of them (Moehring & Boughman, 2019), as seen in two *Gryllus* cricket species that have latent preferences for long trills, which have not evolved yet in any cricket (Gray et al., 2016).

Under the signal-first mechanism, animals may develop preferences quickly after a novel signal evolves. For instance, in the wasp *Nasonia vitripennis*, males evolved a novel pheromone compound and conspecific females are attracted to that compound, but females from a sympatric sister species are not, suggesting that the preference for this compound evolved after it arose within that one lineage (Niehuis et al., 2013). Some have argued, however, that this pattern could also be explained by a secondary loss of preference (Rosenthal, 2017). Theoretically, changes in both signaler and receiver could also occur near simultaneously (Basolo, 1990; reviewed in Broder et al., 2021; Moehring & Boughman, 2019), facilitated by genetic coupling of signals and preferences, whereby the two traits are maintained together through linkage disequilibrium (Alexander, 1962; Hoy et al., 1977; Kronforst et al., 2006; McNiven & Moehring, 2013; Mead & Arnold, 2004; Shaw & Lesnick, 2009; Xu & Shaw, 2019).

**Figure 1.**
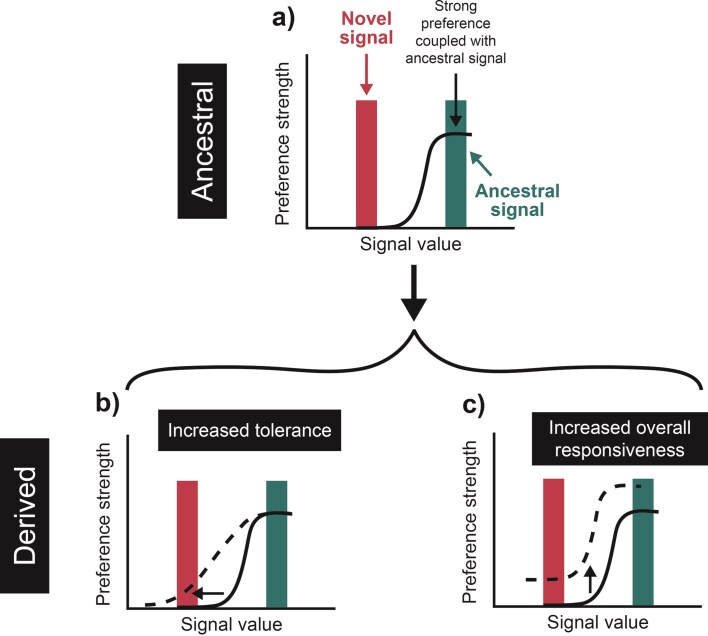
Conceptual figure showing how preferences for novel signal characteristics (like frequency, loudness, and color) might evolve. (a) An open-ended preference function (solid black lines in panels a, b, and c) favors an ancestral signal but discriminates against a novel signal that recently emerged. We illustrate two scenarios that may lead to relaxed preferences in derived populations (dashed black lines in panels b and c). Preference functions could evolve toward (b) increased tolerance to accept novel signals and/or (c) increased overall responsiveness making receivers more prone to respond positively to a broad range of signal values.

Regardless of mechanism, trait-preference models (sensu Kopp et al., 2018) predict a tightly knit statistical relationship between signals and preferences, which raises questions about how novel signals evolve. If signals and preferences are indeed coupled (Figure 1a), when a novel signal trait initially invades (whether through de novo mutation or introgression) and is very rare, almost all receivers should discriminate against it, making the invasion of novel signals challenging to explain. We test an intriguing and underappreciated hypothesis for novel signal evolution that does not rely on the initial tight coupling of signals and preferences and instead builds on the fact that empirical evidence for strong statistical relationships between signals and preferences is rather equivocal (Fowler-Finn & Rodríguez, 2016; Greenfield et al., 2014; Zhou et al., 2011). We hypothesize that relaxed female preferences facilitate the earliest stages of novel signal evolution by allowing such signals to persist long enough for coupled preferences to later evolve (Broder et al., 2021; Moehring & Boughman, 2019; Rowland, 1989). Individuals or populations with relaxed preferences may discriminate less strongly against signals that deviate from the peak preference (have high tolerance or low preference strength; Figure 1b) and/or be highly responsive to a broad range of signals (Figure 1c; sensu Kilmer et al., 2017). Such relaxed preferences could arise through evolutionary (genetic) mechanisms, phenotypic plasticity, or some combination of the two. Regardless of the underlying evolutionary mechanism, females with relaxed preferences should discriminate less against males with novel signal types, allowing novel signals to persist.

Testing mechanisms for how relaxed preferences contribute to signal evolution in a natural context requires a study system in the incipient phases of signal evolution, and where both ancestral and derived populations exist. The Pacific field cricket (*Teleogryllus oceanicus*) presents such an opportunity. Originally from Australia, these crickets were likely transported by Polynesian explorers from French Polynesia to Hawaii around 1500 years ago (Tinghitella et al., 2011; Zhang et al., 2021). In Hawaii, they have recently come into contact with an acoustically orienting parasitoid fly, *Ormia ochracea*. Gravid female flies use male cricket song to find hosts on which to larviposit. The larvae burrow into the crickets and feed upon their tissues, emerging to pupate about 10 days later (Cade, 1975). This strong natural selection has led to the recent emergence of multiple novel signaling types (male morphs) that differ in their song-producing wing structures. Twenty years ago, silent flatwing morphs evolved that produce no audible songs (Zuk et al., 2006). More recently, at least four new male morphs (purring, rattling, curlywing, and smallwing) have evolved that produce distinct but attenuated songs, which help protect males from detection by the parasitoid fly (Gallagher et al., 2022, 2024; Rayner et al., 2019; Tinghitella et al., 2018, 2021).

Have relaxed female preferences accommodated the unusually rapid diversification of male songs in Hawaii? Before engaging in courtship, crickets must first find each other in the large, dark fields where they live. Males attract females from afar using a long-distance calling song (Alexander, 1961) that is particularly important in species recognition (Gray & Cade, 2000; Honda-Sumi, 2005), and without positive female phonotaxis (movement toward song), the two sexes would be unlikely to encounter one another. Females with strong preferences for the typical male signal may never approach novel songs, making proceeding to courtship highly unlikely. Previous work suggests that once they encounter a male, female *T. oceanicus* from Hawaii often accept novel male morphs as mates during one-on-one courtship interactions. For instance, while typical males that sing the original song (hereafter typical males) are almost always mounted at the highest rates, Hawaiian females accept 40%–50% of silent males (Tinghitella & Zuk, 2009), 57% of purring males (Fitzgerald et al., 2022), 60% of rattling males (Gallagher et al., 2024), 60% of curlywing males (Gallagher et al., 2024), and 86%–90% of smallwing males (Gallagher et al., 2024; Zhang et al., 2023). These courtship patterns observed in staged one-on-one interactions lead us to ask whether high acceptance of novel types reflects underlying relaxed preferences and what (mechanistically) precipitates that permissiveness. We thus test for relaxed preferences at the initial, critical stage of mate choice: long-distance mate location.

We ask if relaxed preferences facilitate success of novel songs during long-distance mate attraction. Simultaneously, we test potential underlying mechanisms, which required a geographic approach and paired field- and lab-based experiments. Search costs are a broad lens through which to examine our questions, as high search costs can select for relatively relaxed preferences (Servedio & Bürger, 2014; Yukilevich and Aoki, 2022), and numerous non-mutually exclusive mechanisms may increase search costs. First, *T. oceanicus* has a broad geographic range inclusive of large, panmictic mainland populations in Australia and more recently founded small island populations throughout Oceania. In island populations, search costs are likely high soon after colonization. Kaneshiro (1976) proposed that colonizing isolated islands would select for less discriminating females because particularly choosy individuals may be unlikely to find an acceptable mate (Kaneshiro, 1976, 1980). Second, in Hawaii specifically, relaxed preferences may be favored because the parasitoid fly targets typical males (Gallagher et al., 2022; Rayner et al., 2019; Tinghitella et al., 2021), leaving females with potential mates that are difficult to locate because they have no or drastically attenuated signals (Gallagher et al., 2022) (further enhancing search costs in those populations). Third, relaxed preferences might emerge through plasticity if acoustic environment, recent mating success, population demography, or other factors provide cues about mate availability (search costs) inducing changes in preference shape. Acoustic rearing environment, for instance, impacts female preferences, with females reared in silence being more responsive to male calling song playbacks than females reared hearing preferred songs (Bailey & Zuk, 2008; Rayner et al., 2019; Tinghitella et al., 2021).

We hypothesize that preferences are indeed relaxed in Hawaii relative to elsewhere in the crickets’ range, such that novel songs are more accepted in Hawaii, and that a combination of evolutionary and plastic mechanisms contributes to this pattern. If higher search costs in island populations (e.g., Kaneshiro’s effect) generate relaxed preferences, we expect French Polynesian and Hawaiian populations to be more accepting of males with novel signal types. Subsequent selection in Hawaii due to the parasitoid and related increased search costs in populations with novel male morphs could lead Hawaiian females to be even more accepting of novel types than females from other island populations. If preferences are plastic, we expect to observe differences in female phonotactic behavior between field and lab trials. Of course, plasticity itself may also evolve. Where search costs are high, we may find greater plasticity if that plasticity increases responsiveness to novel songs. Alternatively, plasticity in preferences may be the ancestral state, and a loss of plasticity could facilitate acceptance of novel mates if that loss leads females to have consistently high acceptance of a variety of signals.

## Methods

### Field-based phonotaxis tests

We collected reproductively mature adult female *T. oceanicus* at six field sites: two replicate sites in Queensland, Australia (Cairns and Daintree), in February of 2023, two in French Polynesia (Tahiti and Mo’orea) in December of 2023, and two in Hawaii (Hilo and Wailua) in December of 2022 (Figure S1 and Table S1) using methods that are not biased with respect to song, sex, or life stage. Briefly, rather than locating individuals by sound, we swept by foot in the fields where the crickets are found, collecting animals visually such that all life stages, sexes, and morphs were encountered (following Tinghitella et al., 2018, 2021). Upon collection, we took the animals to local field stations where we housed females in 15-L plastic storage containers with rabbit food ad libitum, egg carton, and cotton with water under natural day–night cycles (at a density of ~30–40 females per container). We acoustically isolated females from calling males for 24 hr before conducting phonotaxis trials.

Our first question was whether female crickets from Australian, French Polynesian, and Hawaiian populations differ in their phonotactic responses to the typical song and newly evolved songs in Hawaii. We addressed this in the field using standardized phonotaxis trials in which we tested each female’s response to the following stimuli: a loop of five purring calling songs (played in immediate succession), a loop of four rattling calling songs, a loop of four typical calling songs, and a silent negative control. All loops were previously used in Gallagher et al. (2022) and Tinghitella et al. (2018, 2021). We randomized the order in which the stimuli were played. We conducted all trials between 2 and 8 hours after sunset (the active period when females search for males) at 24–26 °C and under red light (following Tinghitella et al., 2021). In each trial, we placed a single female cricket under a plastic cup at one end of an arena (50 cm wide × 195 cm long × 25 cm tall) 1 m away from an AOMAIS Sport II Bluetooth speaker that was positioned in the arena’s center. After the female adjusted to the arena, we played the first stimulus track, lifting the cup and allowing her to walk about the arena. The typical *T. oceanicus* loop was broadcast at 70 dBA, the rattling loop at 60 dBA, and the purring loop at 53 dBA (at 1 m away), reflecting biologically realistic amplitudes (Gallagher et al., 2022, 2024; Tinghitella et al., 2018). We confirmed amplitudes using a class 1 PCE-430 sound-level meter set to “A” weighted measurements with fast integration time. We played each stimulus for a maximum of 1 min or until the female contacted the speaker. If the female demonstrated positive phonotactic behavior, but did not contact the speaker in the first minute, we gave her a second minute of playback to allow potential contact. We considered a female to be positively phonotactic when she moved in the direction of the speaker in a classic zigzag pattern without following the wall or circling the arena. In addition to noting whether the female was positively phonotactic, we recorded the maximum distance traveled and the latency to contact the speaker for each stimulus (following Tinghitella et al., 2021). One observer relayed behavioral observations to a recorder while wearing earplugs so they were blind to the stimulus. Given the low levels of phonotaxis exhibited by Australian females to our stimuli, we created a supernormal stimulus (Supplemental Methods) to confirm that females were indeed sexually mature and phonotactic (Figure S2).

### Lab-based phonotaxis tests

To investigate the impacts of environment and experience on female preferences, we repeated our phonotaxis trials in the lab after rearing animals to at least the F2 generation (to avoid transgenerational effects). We reared animals in common garden, but separated by population, in 15-L plastic containers in a clock-shifted, temperature and humidity controlled room set to a 12:12 light:dark cycle and 25 °C. Each 15-L container housed a mixed sex group with access to ad libitum food (Fluker’s Cricket Chow for juveniles and Kaytee rabbit chow for adults), water from moistened cotton, and egg carton. Prior to trials, we haphazardly chose females from each of the six lab-reared populations at the antepenultimate instar, the instar prior to which they develop auditory organs (Kämper, 1992), and isolated them in a sound-insulated incubator inside of 1.89-L containers; thus, females were reared in silence (eliminating song experience as a source of variation in preference behavior). We ensured their virginity at the time of trials and checked isolated females twice a week for eclosion to adulthood. We conducted phonotaxis trials on adult females during their scotoperiod when they were 7–21 days post-eclosion. All equipment and the phonotaxis protocol were identical to the field trials except that we conducted lab-based trials in a temperature-controlled room set to 25 °C. All sample sizes can be found in Table S1.

### Statistical analyses

To address the hypothesis that relaxed preferences facilitate success of novel sexual signals and gain insight into the underlying mechanisms, we first asked how phonotactic behavior depended on region (Australia, French Polynesia, and Hawaii), song type (purring, rattling, typical, and silence), and whether trials were conducted in the field or lab. Using the *lme4* package (Bates et al., 2015) in R Studio version 2024.12.1, we ran a generalized linear mixed model (GLMM) with a binomial distribution in which phonotaxis (yes/no) was the response variable and region, song type, and lab vs. field plus all two-way interactions were included as main effects. Individual ID was a random effect to account for repeated testing of females with the four stimuli. In this model, a significant interaction between region and song type would indicate that there are differences in female preference among regions. As the lab vs. field effect indicates whether preferences are plastic, finding an interaction between region and lab vs. field would indicate that regions differ in the extent to which their preferences are plastic as would occur if plasticity had evolved. This could occur in several ways. For instance, plasticity might be selected for in Hawaii if it facilitates mating where search costs are high, or ancestral plasticity might be lost if it is costly to maintain, leading Hawaiian females to be consistently highly responsive. And, a significant song type by lab vs. field effect would support the hypothesis that plasticity is stimulus dependent.

Next, we constructed preference functions using our field phonotaxis data to examine which preference traits (see below) differed among regions in order to gain insight into the manner in which preferences are relaxed in Hawaii. Preference functions describe signal attractiveness as a function of variation in signaling traits (Neelon et al., 2019) and can take on many shapes (e.g., open-ended or closed) described by quantifying preference traits (Fowler-Finn & Rodríguez, 2012a, 2012b; Kilmer et al., 2017; Neelon et al., 2019; Rodríguez et al., 2006, 2013). We investigated three preference function traits: tolerance (acceptance of trait values that deviate from the peak preference), responsiveness (mean response level across all signal trait values), and strength (how much attractiveness declines as signal values deviate from the peak). An individual with a relaxed preference might, for instance, have high responsiveness, be more tolerant, and/or have low preference strength relative to an individual with a preference strongly coupled to a particular signal (Figure 1). We used PFunc (Kilmer et al., 2017) to visualize the shape of individual and region-level preference functions for the three song types (typical, rattling, and purring songs). Note that because novel songs are categorically different from the typical song, differing in multiple signal traits (e.g., dominant frequency, bandwidth), we visualized preference functions across song types. Preference function traits (tolerance, responsiveness, and preference strength) were extracted from individual-level functions fit to two measures of female responses: phonotaxis (yes/no) and latency to contact. General linear models compared these values across regions.

Having found that Hawaiian females are more responsive to novel songs than those from elsewhere across the crickets’ range, particularly in field studies, we next explored one mechanism, enhanced locomotor behavior, that may underlie that pattern. More locomotory females may be more likely to wander close enough to the quieter purring or rattling males to hear their songs and then pursue them as mates. We asked whether females from different regions differed in the distance they travelled during the silent negative control trials. These data were non-normally distributed, so we ran Kruskal–Wallis tests on distance travelled during silence by region in both the field and lab and conducted pairwise post hoc comparisons with Mann–Whitney–Wilcoxon tests.

Additionally, given that rattling males are only present in Hilo (not Wailua), and purring males are only present in Wailua (not Hilo), we investigated potential local adaptation using a GLMM comparing phonotaxis to these two songs (rattling and purring) between these two populations with female ID as a random effect. If local adaptation has occurred, we expect females to be more responsive to the novel song found in their respective populations. If coevolution with silent flatwing males is critical, Kauai females (where the population was more than 95% silent for at least 60 generations; Bailey et al., 2024) should be the most relaxed of all.

### Model of effective hearing distance

Finally, because the pattern of female responses to purring and rattling songs across the crickets’ range differed, with phonotaxis to purring being rare overall (see results), we developed a computational model to determine from how far away female *T. oceanicus* are likely to be able to hear and use purring, rattling, and typical songs in the context of long-distance mate location. Using previously published neural audiogram data in *T. oceanicus* (Atkins & Pollack, 1986; Fullard et al., 2010) and the sound intensity levels and peak frequencies of different types of cricket calling songs, we built a model estimating effective hearing distances for each song type. We based the model on peak frequencies of the purring, rattling, and typical songs reported in Gallagher et al. (2022) and sound intensity levels from Wikle et al. (2025), modeling responses to the average dominant frequency ± 1 *SD* and the average amplitude ± 1 *SD*. The model accounts for non-frequency-dependent damping of sound with distance and frequency-dependent attenuation due to atmospheric absorption. Detailed modeling methods can be found in Wikle et al. (2025) where we previously modeled the effective hearing distances to these songs for the parasitoid fly. There is substantial inter-individual variation in spectral characteristics (dominant frequency and bandwidth) and amplitude of novel songs (Gallagher et al., 2023). All of these likely influence effective hearing distances, and our model examines two important axes of variation (amplitude and dominant frequency) that characterize differences among song types, but we are unfortunately not able to account for variation in bandwidth in this model.

Several previously published neural audiograms based on ON1 (omega neuron 1—responsible for directional processing of cricket songs) recordings from *T. oceanicus* have been collected from lab-reared animals that originated from Australia (Atkins & Pollack, 1986; Fullard et al., 2010; Kostarakos et al., 2009) and Mo’orea (Fullard et al., 2010). Since purring and rattling songs contain frequencies that extend well above 10 kHz (Gallagher et al., 2022; Tinghitella et al., 2021), we digitized neural audiograms from Atkins and Pollack (1986) and Fullard et al. (2010), both of which describe auditory tuning of ON1 up to 40 kHz (whereas Kostarakos et al., 2009, only examines neural responses from 3 to 6 kHz). We used Meazure 2.0 (written by Baron Roberts, C Thing Software; Roberts, 2013) to capture data points across three audiograms. As the neural audiograms from these two studies were conducted for different frequency sampling points, we imported the captured data into MATLAB, used linear interpolation between frequency sampling points, and averaged across the three interpolated audiograms to produce an average *T. oceanicus* audiogram (Figure S3). We determined thresholds for song peak frequencies from the interpolated average neural audiogram. Following the approach described in Wikle et al. (2025), we calculated attenuation curves over distance starting at peak amplitude for nine different frequency and amplitude combinations for each song type and determined the distance at which a particular song sound pressure level reached the threshold identified from the average neural audiogram required to elicit a neural response. We calculated these estimated hearing distances for each song type.

## Results

**Figure 2.**
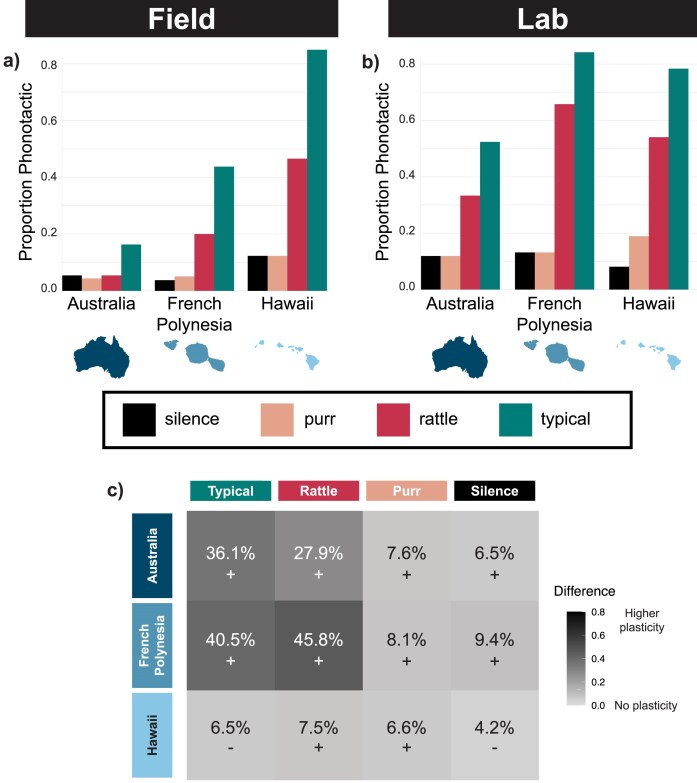
Field (a) and lab (b) phonotaxis data showing the proportion of female crickets that were positively phonotactic to silence (black), purr (light salmon), rattling (deep pink), and typical (teal) songs in each region (Australia, French Polynesia, and Hawaii). In the field, females exhibited the highest levels of phonotaxis to the novel rattling and typical songs in Hawaii, intermediate levels in French Polynesia, and low levels in Australia. In the lab, there were no significant differences in phonotaxis to each song among regions. (c) Heat map showing the difference in the proportion of phonotactic females in the field and lab for each region and song type. Darker shades indicate larger differences between the lab and field settings, and + and − signs indicate the direction of that difference.

Female *T. oceanicus* exhibited a consistent rank order of song preferences regardless of whether they were tested in the field or lab (GLMM song: *p* = 8.90e−05; Table 1 and Figure 2). Typical song was preferred over the novel rattling song, which was preferred over purring and silence (purring and silence did not differ from one another; Table S2). Additionally, as expected, females from Hawaii were most accepting (Table 1; GLMM region: *p* = 0.0009), and preferences were indeed plastic (Table 1; GLMM lab vs. field: *p* = 0.003). However, we found intriguing differences in the shape of preferences among regions (GLMM region × song: *p* = 0.0002; Table 1). Phonotactic responses to typical and rattling songs depended on which region females were from, but responses to the silent negative control and purring songs were universally low and did not differ among regions (all pairwise *p* > 0.05). Regarding the novel rattling song, Hawaiian females were more phonotactic than French Polynesian and Australian females, whose phonotactic responses did not differ significantly from one another. Regarding typical song, Hawaiian females were again the most phonotactic, followed by French Polynesia, and then Australia. For full pairwise results, see Table S2.

**Table 1.** Outputs of GLMM addressing phonotaxis (yes/no) in the field and lab.

Effects	χ²	df	*p*
Region	14.067	2	**0.0009**
Lab vs. field	9.012	1	**0.003**
Song	21.351	3	**8.90e−05**
Region × lab vs. field	16.792	2	**0.0002**
Region × song	26.290	6	**0.0002**
Lab vs. field × song	6.104	3	0.107

*Note*. GLMM = generalized linear mixed model. Bold *p*-values are significant at < 0.05.

Phonotactic responses differed between the field and lab experiments (were plastic), suggesting that experience in nature strongly influences phonotactic behavior. We found that plasticity in preferences is region specific (GLMM lab vs. field × region: *p* = 0.0002; Table 1). To better visualize the detailed differences in behavior between lab and field experiments (plasticity), we created a heat map (Figure 2c) where darker shades illustrate regions and song types for which phonotactic behavior differed greatly across contexts (lab vs. field) and lighter shades indicate little change in behavior across contexts. We took the absolute value of the difference in proportion phonotactic in the field and lab. The heat map illustrates that the differences in phonotactic behavior between the lab and field were most pronounced for females from Australia and French Polynesia and with respect to rattling and typical songs (Figure 2c; see also pairwise differences in Table S2). Both were much more phonotactic to rattling and typical songs in the lab than field. In contrast, Hawaiian females exhibited little plasticity and were consistently highly responsive to the rattling and typical songs (Figure 2a–c); in other words, Hawaiian females always show broadly permissive preferences regardless of changes in the environment between lab and field.

**Figure 3.**
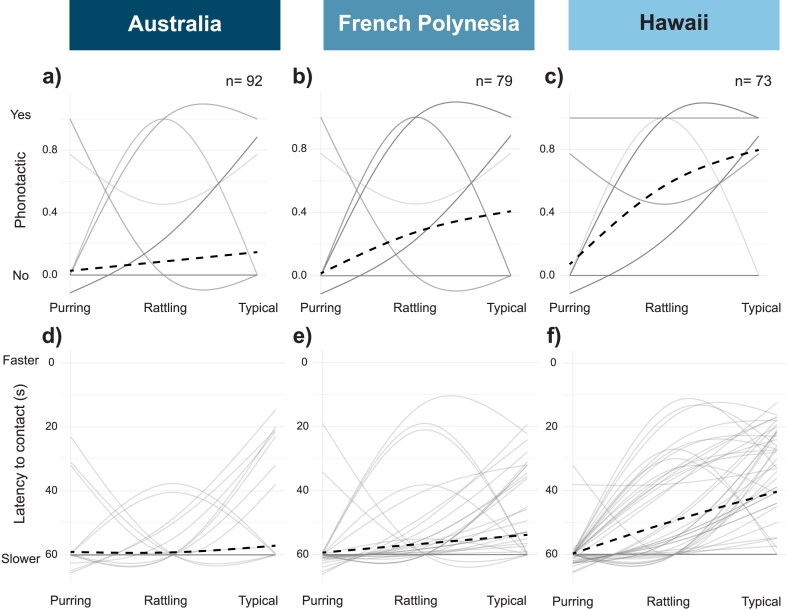
Preference functions of Australian, French Polynesian, and Hawaiian females generated from behavioral responses to purring, rattling, and typical songs. Panels a–c show whether females displayed phonotactic behavior (yes/no) and panels d–f show how quickly females contacted the speaker broadcasting songs. Note that the y-axis for latency to contact (d–f) runs from 60s to 0s so that behavioral responses indicating stronger preference are always located at the top of the y-axis for ease of interpretation. Gray lines show individual-level splines fit by the program Pfunc to female responses and black lines indicate the average preference function at the level of the region. As identical splines are possible with binomial responses, many replicates overlap in panels a–c; sample sizes are indicated for each region. Color (gray) intensity reflects the number of individuals with the same preference function shape, with darker grays indicating more individuals.

We then asked which specific preference function traits (tolerance, strength, and/or responsiveness) differed among regions to better understand what is “relaxed” about preferences in Hawaii by generating individual and region-level preference functions for females from each region with respect to the purring, rattling, and ancestral stimuli. When examining whether females were phonotactic or not and how quickly they contacted stimuli, responsiveness, tolerance, and preference strength differed among regions (Figure 3 and Table 2). Hawaiian females were more responsive overall, approaching a greater proportion of all three song types and doing so more quickly, but interestingly, were also less tolerant and had higher preference strength (Table 2). The latter two patterns seem to stem from the rather low phonotaxis rates of Australian females to the typical *T. oceanicus* song in the field, though, recall that we did confirm these females were capable of phonotaxis by testing their behavior with a supernormal stimulus (Figure S2). The very high rates of phonotaxis to typical songs in Hawaii generate steeper preference functions for Hawaiian females, less steep functions for French Polynesian females, and nearly flat functions for Australian females, reflecting their overall low rates of phonotaxis. Incidentally, we also generated preference functions for each female and region with respect to the mean dominant frequency (one axis of song variation) of the purring, rattling, and ancestral songs, with qualitatively identical results (e.g., all preference traits differ between regions, with Hawaiian females being consistently more responsive than those from other regions; Table S3).

**Table 2.** Regional differences in female cricket responsiveness, tolerance, and preference strength in response to *T. oceanicus* song types.

Model name and type	*F* value	df	*p*
A. Phonotaxis yes/no			
Responsiveness	59.836	2	**<2.2e−16**
Tolerance	16.525	2	**1.88e−07**
Preference strength	10.832	2	**3.13e−05**
B. Latency to contact			
Responsiveness	32.930	2	**3.77e−13**
Tolerance	20.440	2	**7.86e−09**
Preference strength	15.263	2	**6.53e−07**

*Note*. Preference function traits were extracted from individual-level functions fit to two measures of female responses: phonotaxis (yes/no) and latency to contact. Regional differences in these parameters were tested using GLMs. Bold *p*-values are significant at < 0.05.

**Figure 4.**
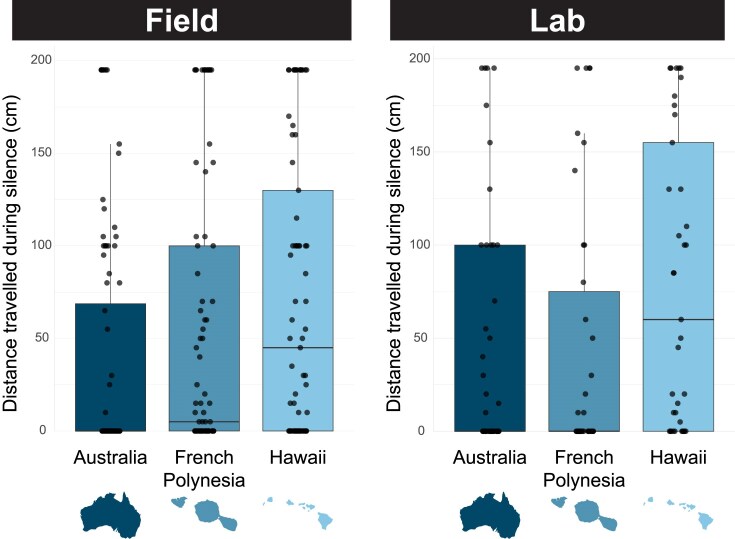
Box plots showing distance travelled during silence across regions, representing baseline levels of locomotory behavior. Hawaiian females travel more than Australian females in both field (left) and lab (right) environments.

We also ran two post hoc analyses to investigate potential mechanisms that may underlie the relaxed preferences in Hawaii. Both Hawaiian (HI) and French Polynesian (FP) females travelled more than Australian (AU) females in the field (Figure 4; χ^2^ = 15.27, df = 2, *p* = 0.0005; pairwise values: AU–FP *p* = 0.009, AU–HI *p* = 0.0002, FP–HI *p* = 0.155). And in the lab, Hawaiian females travelled more than French Polynesian and Australian females (χ^2^ = 7.128, df = 2, *p* = 0.028; pairwise values: AU–FP *p* = 0.677, AU–HI *p* = 0.034, FP–HI *p* = 0.016). Additionally, we tested for potential local adaptation of phonotaxis in Hawaii whereby females are more responsive to the novel song (purring or rattling) found in their respective populations (Wailua or Hilo), but found no such patterns (GLMM: song *p* = 0.0003, population *p* = 0.509, song × population *p* = 0.143).

**Figure 5.**
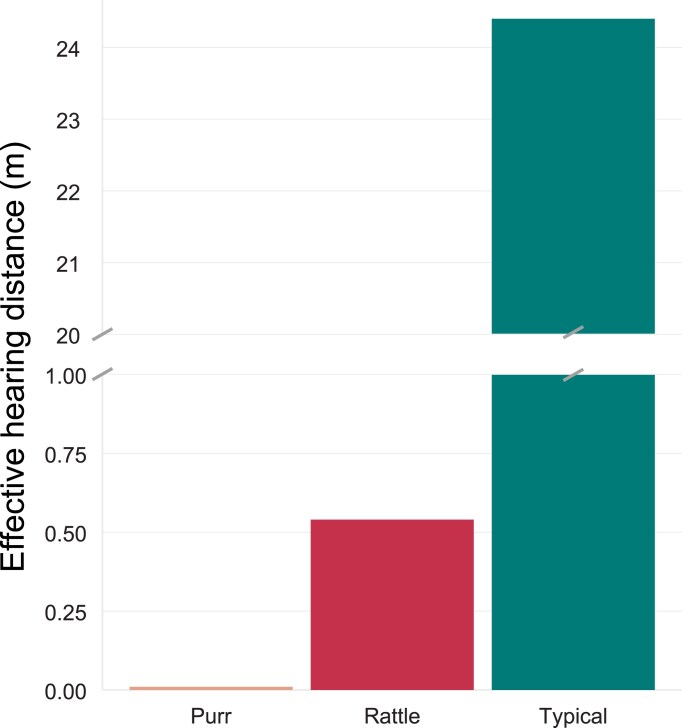
Modeled distance at which female *T. oceanicus* from Moorea and Australia can hear the typical and novel (purring and rattling) songs. Note that the y-axis is broken to facilitate viewing of the dramatic differences in effective hearing distance between morphs.

Finally, we developed a computational model to estimate the distances at which female *T. oceanicus* from Australia and French Polynesia should be able to hear and use the purring, rattling, and typical calling songs. A model of mean peak frequencies and amplitudes among each song type revealed dramatic differences in effective hearing distances across song types (Figure 5). Estimated hearing distances for purring were less than 10 cm, whereas rattling should be detectable 0.54 m away, and typical song 24.4 m away (Table S4; Figure 5).

## Discussion

Conspicuous male sexual signals are often tightly coupled with receiver preferences via coevolutionary processes. However, tight coupling may make it difficult for novel signals to emerge if females discriminate strongly against signals that differ from the ancestral. We asked whether relaxation of female preferences facilitates the earliest stages of novel signal evolution, integrating studies of long-distance phonotaxis conducted in the field and lab and over a broad geographic range to investigate mechanisms that may lead to relaxed preferences. Consistent with the hypothesis that relaxed preferences facilitate novel signals, in the field, females from Hawaiian populations exhibited relaxed preferences relative to those from elsewhere in their range, accepting a much larger proportion of males who sing the newly evolved rattling song (Figure 2a). Only 5.4% of females from Australia, for instance, were phonotactic to rattling song, compared to 46.6% in Hawaii. This suggests rattling would have been more strongly selected against in Australia, had it emerged there. In the lab, where we eliminated environmental differences by rearing individuals and conducting phonotaxis trials in common garden, there were no differences in phonotactic responses among regions. This change was driven largely by increased phonotaxis of French Polynesian and Australian females in the lab (Figure 2b and c). In contrast, rates of phonotaxis remained high for Hawaiian females regardless of variation in environment (rearing and testing conditions in the field or lab; Figure 2c). Thus, the relaxation of preferences in Hawaii may stem from the evolutionary loss of plasticity in derived Hawaiian populations, as discussed below.

We set out to examine whether and how relaxed preferences during mate location might facilitate the evolution of two new and novel signals, purring and rattling. Yet, we only find that preferences for the rattling and typical songs differ across regions. While Hawaiian females were significantly more phonotactic to the novel rattling song and the typical song than females from elsewhere (Figure 2a) in field trials, phonotaxis to the second novel song (purring) did not differ among regions and responses to it were universally low across the crickets’ range. Our computational model provides important context here. Purring songs are one of the most broadband and quiet novel songs that have evolved in Hawaii (Gallagher et al., 2022; Tinghitella et al., 2018). Our model suggests that female *T. oceanicus* are unlikely to be able to hear these songs at distances of even 10 cm away. If so, purring is unlikely to be used in traditional long-distance mate location and may only be an effective signal at much shorter distances. Females may employ other tactics (such as enhanced locomotory behavior; Balenger & Zuk, 2015; Tinghitella et al., 2009) to find purring males and then exert preferences in short-distance courtship interactions.

Two caveats are important here. First, the available neural audiograms for *T. oceanicus* only exist for Australian and French Polynesian animals, and were conducted more than 15 years ago (Atkins & Pollack, 1986; Fullard et al., 2010). There are no available neural or behavioral audiograms for Hawaiian populations. If *T. oceanicus* hearing has evolved in Hawaii in ways that facilitate location of novel male songs, as the hearing of the parasitoid fly has (Wikle et al., 2025), our model may underestimate distances at which novel songs can be heard. Indeed, previous work shows that purring songs can be detected by some Hawaiian females at 1 m away (Tinghitella et al., 2018, 2021). Second, there are some limitations to our modeling approach, in that the model considers detection of cricket calling songs across varied dominant frequencies and amplitudes but does not account for variation in bandwidth. The novel purring and rattling songs are substantially more broadband than the typical *T. oceanicus* song (purring particularly; Gallagher et al., 2022; Tinghitella et al., 2018). A single dominant frequency is likely not representative of the frequency content female crickets are responding to within broadband songs; thus, accounting for variation in bandwidth would provide a more nuanced understanding of the effective hearing distance of novel songs. Nevertheless, the dramatically shorter distances over which purring is audible to females could indicate different roles of calling songs for purring and rattling individuals. In other words, purring may not be effective in long-distance mate location, leading to the lack of differences across regions we find here.

Differences in phonotactic behavior across regions and between lab and field contexts contribute to our understanding of the mechanisms that may have led to relaxed mating preferences in Hawaii. Notably, we saw differences in phonotaxis between females from different regions in field-based trials, but not lab-based trials. In the field, Hawaiian females were more phonotactic (Figure 2a) to typical and rattling song than Australian and French Polynesian females, consistent with high search costs and Kaneshiro’s effect and perhaps more recent selection in Hawaii. When reared in common garden in the lab, females from all regions exhibited similar levels of phonotaxis to all stimuli (Figure 2b). This difference between lab and field appears driven by increased phonotaxis to typical and rattling songs by females from Australia and French Polynesia in the lab environment (Figure 2c). One compelling interpretation of this pattern is that Hawaiian females respond very positively to the typical song and novel rattling song across broad experience and environmental contexts because they have lost ancestral plasticity in preferences. Indeed, while Australian females show plasticity in that they only approach novel signals when reared in the lab (in silence), Hawaiian females exhibit phonotaxis to novel signals regardless of rearing environment and experience (e.g., lab or field). Hawaiian females are therefore less plastic than Australian and French Polynesian populations.

What might differ in field and lab settings that leads to the plastic phonotactic responses of Australian and French Polynesian, but not Hawaiian, females? In nature, *T. oceanicus* populations likely differ in numerous factors that could impact the strength of mating preferences. For instance, variation in demography (e.g., adult density and sex ratios), acoustic environments, body condition, mating experience, age, predation risk, injury and infection, and genetic diversity is likely across populations, and these factors seem particularly relevant to reproductive behavior (Bailey, 2011; Bailey & Zuk, 2008; Bailey et al., 2010; Bauer-Nilsen et al., 2025; Cotton et al., 2006; Hedrick & Dill, 1993; Jennions & Petri, 1997; Lierheimer & Tinghitella, 2017; Richardson & Zuk, 2023; Tinghitella et al., 2013). Some of these factors may contribute to the differences we observed between field and lab individuals, and we discuss a few likely possibilities here. For instance, there are reasons to believe that individuals in poor condition might show either reduced or increased investment in costly mate choice (depending on whether they are too compromised to invest or are making a terminal investment, respectively; Richardson et al., 2025). Similarly, theory predicts that virgin females should be less choosy than mated females as they risk dying unmated (Kokko & Mappes, 2005). Given that field-collected individuals in the experiment were unlikely to be virgins and lab individuals were all virgins, this could explain some observed trends.

Acoustic environment has been shown to be particularly important for inducing plasticity in this study system, and it differs across the populations we studied and between lab and field settings (Bailey, 2011; Bailey & Zuk, 2008; Bailey et al., 2010). For instance, in Australia and French Polynesia, females only experience the ancestral song. Under these circumstances, it would be advantageous for females to be more discriminating when males are abundant and less discriminating when search costs are higher and males are rare (Kokko & Mappes, 2005; Real, 1990). Such plasticity likely facilitated the colonization of very remote islands like those in Hawaii (Ghalambor et al., 2007). More recently, though, the acoustic environment in Hawaii varies—females may hear little song at all (e.g., Wailua where the population is primarily purring and historically silent; Gallagher et al., 2023; Zuk et al., 2018) or a large proportion of novel song (e.g., Hilo where the population contains typical, rattling, smallwing, and curlywing; Gallagher et al., 2024), both of which mimic low preferred mate availability. We hypothesize that if maintaining plasticity is costly, selection may have favored its loss under these conditions. In the lab setting, all females were reared in acoustic isolation, and Australian and French Polynesian females responded by enhancing their phonotactic responses, even to song types they had never heard before, yet Hawaiian females retained their broad preferences regardless. Given that the silent lab environment more closely resembles acoustic environments experienced by Hawaiian females than it does Australian or French Polynesian females, we also reared Wailua females in a lab environment in which they heard all Hawaiian songs (Figure S4). If there was no loss in plasticity, we would expect those Wailua females to become choosier when reared in song. However, we instead found no differences in the behavior of Wailua females reared in silence versus song, supporting our contention that the loss of ancestral plasticity at least partly explains the universally high acceptance of novel males by Hawaiian females (regardless of environment and experience).

Given the collective evidence presented above, we hypothesize that ancestral plasticity likely allowed colonizing *T. oceanicus* females to successfully reproduce in newly founded Hawaiian populations, but that plasticity was later lost evolutionarily, perhaps because it was costly. If so, genetic accommodation could result in females in Hawaii having consistently high responsiveness to varied sexual signals (relaxed preferences relative to ancestral populations). Relaxed preferences could then facilitate the evolution of novel male songs when they arose. Given the important role of plasticity in colonization and the strong selection that female preferences impose on animal signals, future research should untangle these potential impacts.

Multiple different preference function shapes may characterize responses of females with relaxed preferences. An increase in tolerance, increase in overall responsiveness, and/or a reduction in the strength of preference for the ancestral signal (relative to novel signal space) can all lead to more relaxed preferences (Figure 1; Kilmer et al., 2017). Interestingly, while all these characteristics differed statistically across regions in field-based analyses, only responsiveness differed in the direction expected in Hawaii (Figure 3 and Table 2). Females in Hawaii were more responsive (increased height of the preference function) to all stimuli. However, they were actually less tolerant and had higher preference strength. These two patterns are likely driven by an increase in preference for typical song in Hawaii, and reduced discrimination between the ancestral song and the novel songs, relative to females from other regions. It appears enhanced responsiveness alone may be sufficient to facilitate novel signal evolution.

We also investigated whether increased locomotory behavior might enhance the likelihood of Hawaiian females contacting novel songs. We have regularly observed that, anecdotally, both males and females appear to walk on top of the grass more in Hawaii than they do elsewhere in the Pacific, which leads us to hypothesize that mate location occurs, at least partly, through random walking encounters in Hawaii. We compared the distance travelled by females from the three regions during silence and found that, indeed, Hawaiian females are more locomotory than Australian females, for both field-reared and lab-reared individuals (Figure 4). This is a particularly intriguing pattern in light of work that suggests more exploratory individuals may be better invaders of new habitats (Cote et al., 2010; Damas-Moreira et al., 2019; Wright et al., 2010), and suggests that selection has favored the consistently high levels of locomotory behavior in Hawaii. It would be interesting to know whether other behavioral traits, like personality, differ across *T. oceanicus* populations and whether this facilitates the evolution of novel songs. Interestingly, though, within Hawaii, we did not see differences in phonotaxis to rattling and purring between the Hilo and Wailua populations. Given that each of these populations contains only one of the novel morphs (rattling in Hilo and purring in Wailua), we hypothesized that there might be local adaptation to detect and respond positively to the local novel song. Instead, both populations are equally phonotactic to the two songs, suggesting the loss of plasticity and high overall responsiveness in Hawaii is a more important contributor to the success of novel songs at this time.

Finally, our finding that differences in phonotaxis among regions are largely plastic differs from previous findings for courtship behavior in the same species (Tinghitella & Zuk, 2009). That study, conducted shortly after the silent morph evolved, revealed that ancestral Australian females discriminated against silent males more strongly than island females in common garden lab-based settings (Tinghitella & Zuk, 2009); thus, the relaxed mating requirements of *T. oceanicus* during courtship are evolved differences. Of course, there may also be plasticity in courtship. This difference suggests that phonotaxis preferences might be more plastic than mating decisions during courtship, and future work should investigate female courtship responses to the novel songs across regions and in both lab and field settings.

Whether novel signals emerge through signaler or receiver first mechanisms is a classic chicken and egg scenario in evolutionary biology and animal communication. In many systems, we are stuck looking back and will never truly know which came first. This study capitalizes on the rare opportunity to catch novel signal evolution in real time and still have access to ancestral populations that have not evolved with the novel signals. Our results suggest that perhaps neither strong preferences nor signals evolve first, but rather relaxed female preferences are a first step (Figure 1). This is especially clear in the French Polynesia field results where females exhibit more relaxed preferences (accept more novel signals) than females in Australia, despite no males with novel signals existing in French Polynesia. If preferences were relaxed through any of the mechanisms described here, it would be easier for a population to climb a fitness peak toward novel sexual signals like those we have observed. Our results suggest that relaxed preferences evolved in Hawaii through a loss of ancestral plasticity, setting the stage for novel signals to then spread rapidly in Hawaii. Our results provide insight into how novel signals gain a foothold when they initially invade, after which coupled preferences may eventually evolve, perhaps leading to reproductive isolation. Alternatively, relaxed preferences may remain longer term, facilitating the maintenance of signal diversity within populations.

## Supplementary Material

qraf047_Supplemental_File

## Data Availability

Data and R code files can be found on Dryad (https://doi.org/10.5061/dryad.t76hdr8ds).
