# Supplementary material for: How relaxed preferences facilitate the evolution of novel animal signals
Source: Evol Lett. 2025 Dec 8;10(1):104–17. doi: 10.1093/evlett/qraf047 (PMC12870860; doi:10.1093/evlett/qraf047)
Supplement: qraf047_Supplemental_File [file qraf047_supplemental_file.docx]

Supplement - Welsh et al.

**Table S1.** Field site coordinates, collection dates, and phonotaxis sample sizes in lab and field.

| **Site** | **Longitude** | **Latitude** | **Field Sample Size** | **Lab Sample Size** | **Collection Dates** |
| --- | --- | --- | --- | --- | --- |
| Cairns | -16.809368 | 145.686621 | 60 | 17 | February 2023 |
| Daintree | -16.25067 | 145.322304 | 32 | 25 | February 2023 |
| Tahiti | -17.5707 | -149.61834 | 40 | 20 | December 2023 |
| Mo’orea | -17.490344 | -149.82634 | 40 | 18 | December 2023 |
| Hilo | 19.7039066 | -155.0801 | 40 | 16 | December 2022 |
| Wailua | 22.0671513 | -159.39583 | 33 | 21 | December 2022 |

**Table S2.** Pairwise p-values for the three 2-way interactions from the GLMM. Aus=Australia, FP=French Polynesia, and HI= Hawaii.

| **region*lab vs field** | | **region*song** | | **region *song (cont.)** | | **song*lab vs field** | |
| --- | --- | --- | --- | --- | --- | --- | --- |
| **pairwise** | **p** | **pairwise** | **p** | **pairwise** | **p** | **pairwise** | **p** |
| Aus field - Aus lab | 0.0001 | Aus purr - FP purr | 1.000 | Aus rattle - FP silence | 0.868 | field purr - lab purr | 0.196 |
| FP field - FP lab | <0.0001 | Aus purr - HI purr | 0.834 | Aus rattle - HI silence | 0.995 | field purr - field rattle | 0.0003 |
| HI field - HI lab | 1.000 | Aus purr - Aus rattle | 0.640 | Aus rattle - Aus typical | 0.074 | field purr - lab rattle | <.0001 |
| Aus field - FP field | 0.233 | Aus purr - FP rattle | <.0001 | Aus rattle - FP typical | <.0001 | field purr - field silence | 1.000 |
| Aus field-HI field | <0.0001 | Aus purr - HI rattle | <.0001 | Aus rattle - HI typical | <.0001 | field purr - lab silence | 0.779 |
| Aus field-FP lab | 0.0001 | Aus purr - Aus silence | 1.000 | FP rattle - HI rattle | 0.977 | field purr - field typical | <.0001 |
| Aus field-HI lab | <0.0001 | Aus purr - FP silence | 1.000 | FP rattle - Aus silence | <.0001 | field purr - lab typical | <.0001 |
| FP field-HI field | <0.0001 | Aus purr - HI silence | 1.000 | FP rattle - FP silence | <.0001 | lab purr - field rattle | 0.924 |
| FP field-Aus lab | 0.144 | Aus purr - Aus typical | 0.0001 | FP rattle - HI silence | 0.0001 | lab purr - lab rattle | <.0001 |
| FP field-HI lab | 0.0017 | Aus purr - FP typical | <.0001 | FP rattle - Aus typical | 0.953 | lab purr - field silence | 0.276 |
| HI field-Aus lab | 0.341 | Aus purr - HI typical | <.0001 | FP rattle - FP typical | 0.010 | lab purr - lab silence | 0.984 |
| HI field-FP lab | 0.978 | FP purr - HI purr | 0.969 | FP rattle - HI typical | <.0001 | lab purr - field typical | <.0001 |
| Aus lab-FP lab | 0.160 | FP purr - Aus rattle | 0.936 | HI rattle - Aus silence | <.0001 | lab purr - lab typical | <.0001 |
| Aus lab-HI lab | 0.640 | FP purr - FP rattle | <.0001 | HI rattle - FP silence | <.0001 | field rattle - lab rattle | <.0001 |
| FP lab-HI lab | 0.970 | FP purr - HI rattle | <.0001 | HI rattle - HI silence | <.0001 | field rattle - field silence | 0.0005 |
|  |  | FP purr - Aus silence | 1.000 | HI rattle - Aus typical | 0.180 | field rattle - lab silence | 0.409 |
|  |  | FP purr - FP silence | 1.000 | HI rattle - FP typical | 0.646 | field rattle - field typical | <.0001 |
|  |  | FP purr - HI silence | 1.000 | HI rattle - HI typical | <.0001 | field rattle - lab typical | <.0001 |
|  |  | FP purr - Aus typical | 0.003 | Aus silence - FP silence | 1.000 | lab rattle - field silence | <.0001 |
|  |  | FP purr - FP typical | <.0001 | Aus silence - HI silence | 1.000 | lab rattle - lab silence | <.0001 |
|  |  | FP purr - HI typical | <.0001 | Aus silence - Aus typical | 0.0002 | lab rattle - field typical | 0.999 |
|  |  | HI purr - Aus rattle | 1.000 | Aus silence - FP typical | <.0001 | lab rattle - lab typical | 0.003 |
|  |  | HI purr - FP rattle | 0.002 | Aus silence - HI typical | <.0001 | field silence - lab silence | 0.889 |
|  |  | HI purr - HI rattle | <.0001 | FP silence - HI silence | 1.000 | field silence - field typical | <.0001 |
|  |  | HI purr - Aus silence | 0.930 | FP silence - Aus typical | 0.002 | field silence - lab typical | <.0001 |
|  |  | HI purr - FP silence | 0.920 | FP silence - FP typical | <.0001 | lab silence - field typical | <.0001 |
|  |  | HI purr - HI silence | 0.996 | FP silence - HI typical | <.0001 | lab silence - lab typical | <.0001 |
|  |  | HI purr - Aus typical | 0.124 | HI silence - Aus typical | 0.009 | field typical - lab typical | 0.0006 |
|  |  | HI purr - FP typical | <.0001 | HI silence - FP typical | <.0001 |  |  |
|  |  | HI purr - HI typical | <.0001 | HI silence - HI typical | <.0001 |  |  |
|  |  | Aus rattle - FP rattle | 0.003 | Aus typical - FP typical | 0.0001 |  |  |
|  |  | Aus rattle - HI rattle | <.0001 | Aus typical - HI typical | <.0001 |  |  |
|  |  | Aus rattle - Aus silence | 0.809 | FP typical - HI typical | 0.154 |  |  |

**Table S3.** Regional differences in female cricket responsiveness, tolerance, and preference strength in response to *T. oceanicus* song types. Preference function traits were extracted from individual-level functions fit to two measures of female responses: phonotaxis (yes/no) and latency to contact with respect to the mean dominant frequency (one axis of song variation) of the purring, rattling, and ancestral songs. Regional differences in these parameters were tested using GLMs.

| **Model Name & Type** | **F value** | **df** | **p** |
| --- | --- | --- | --- |
| A. Phonotaxis yes/no |  |  |  |
| Responsiveness | 58.845 | 2 | **<2.2e-16** |
| Tolerance | 21.666 | 2 | **2.27e-09** |
| Preference strength | 10.72 | 2 | **3.49e-05** |
| B. Latency to contact |  |  |  |
| Responsiveness | 31.681 | 2 | **9.61e-13** |
| Tolerance | 19.726 | 2 | **1.42e-08** |
| Preference strength | 14.796 | 2 | **9.78e-07** |

**Table S4.** Estimated effective hearing distance (mean +/- SD) of Australia and Mo’orea Pacific field crickets for typical, rattling, and purring songs. For each song, we modeled variation in amplitude at 10 cm distance and peak frequency using the mean value and +/- 1 standard deviation (SD; from Gallagher et al. 2022). See details in Methods. Note that the bolded cell in the center of each table (estimated hearing distance at mean amplitude and mean peak frequency) shows the values that are plotted in Figure 5.

| **Typical effective hearing distance (m)** | | Amplitude in dB at 10cm | | |
| --- | --- | --- | --- | --- |
|  |  | -1 SD (82.82) | mean (92) | +1 SD (96.38) |
| Peak frequency (kHz) | -1 SD (4686) | 7.56 | 21.75 | 36 |
|  | mean (4877) | 8.48 | **24.40** | 40.4 |
|  | +1 SD (5069) | 7.56 | 21.75 | 36 |
| **Rattling effective hearing distance (m)** | | Amplitude in dB at 10cm | | |
|  |  | -1 SD (69.29) | mean (73.77) | +1 SD (76.73) |
| Peak frequency (kHz) | -1 SD (3179) | Not detectable at < 10 cm | Not detectable at < 10 cm | Not detectable at < 10 cm |
|  | mean (5806) | 0.32 | **0.54** | 0.75 |
|  | +1 SD (7509) | 0.15 | 0.24 | 0.34 |
| **Purring effective hearing distance (m)** | | Amplitude in dB SPL at 10 cm | | |
|  |  | -1 SD (49.35) | mean (53.71) | +1 SD (56.62) |
| Peak frequency (Hz) | -1 SD (5263) | 0.1 | 0.15 | 0.21 |
|  | mean (9205) | < 10 cm | **< 10 cm** | < 10 cm |
|  | +1 SD (13147) | < 10 cm | < 10 cm | < 10 cm |

**
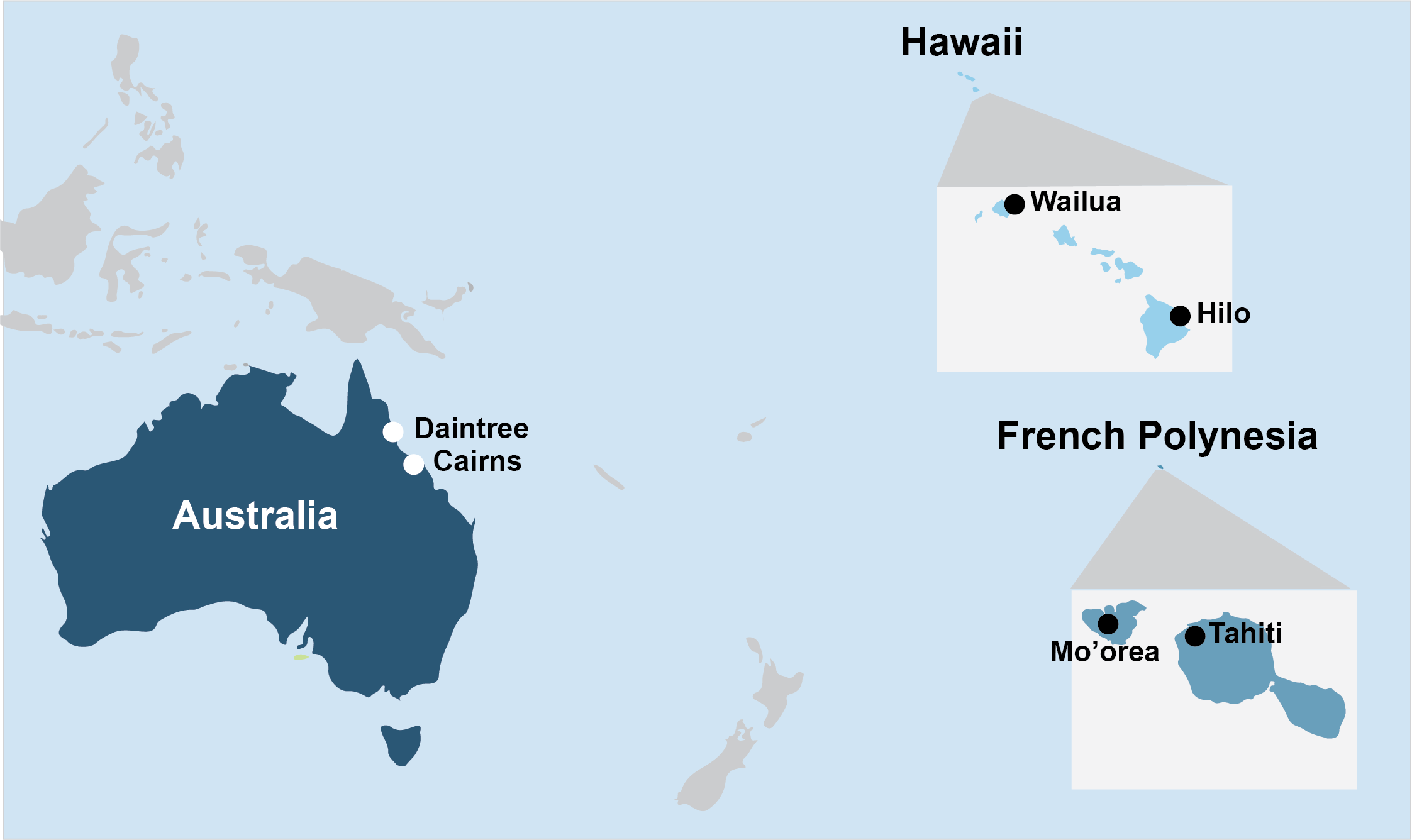
**

**Figure S1.** Map of field sites. We conducted field trials at two replicate field sites in Australia (Daintree and Cairns), two in French Polynesia (Tahiti and Mo’orea), and two in Hawaii (Wailua and Hilo). We also collected eggs from these sites and returned them to the lab where we performed a second set of phonotaxis trials after rearing in common garden.

**
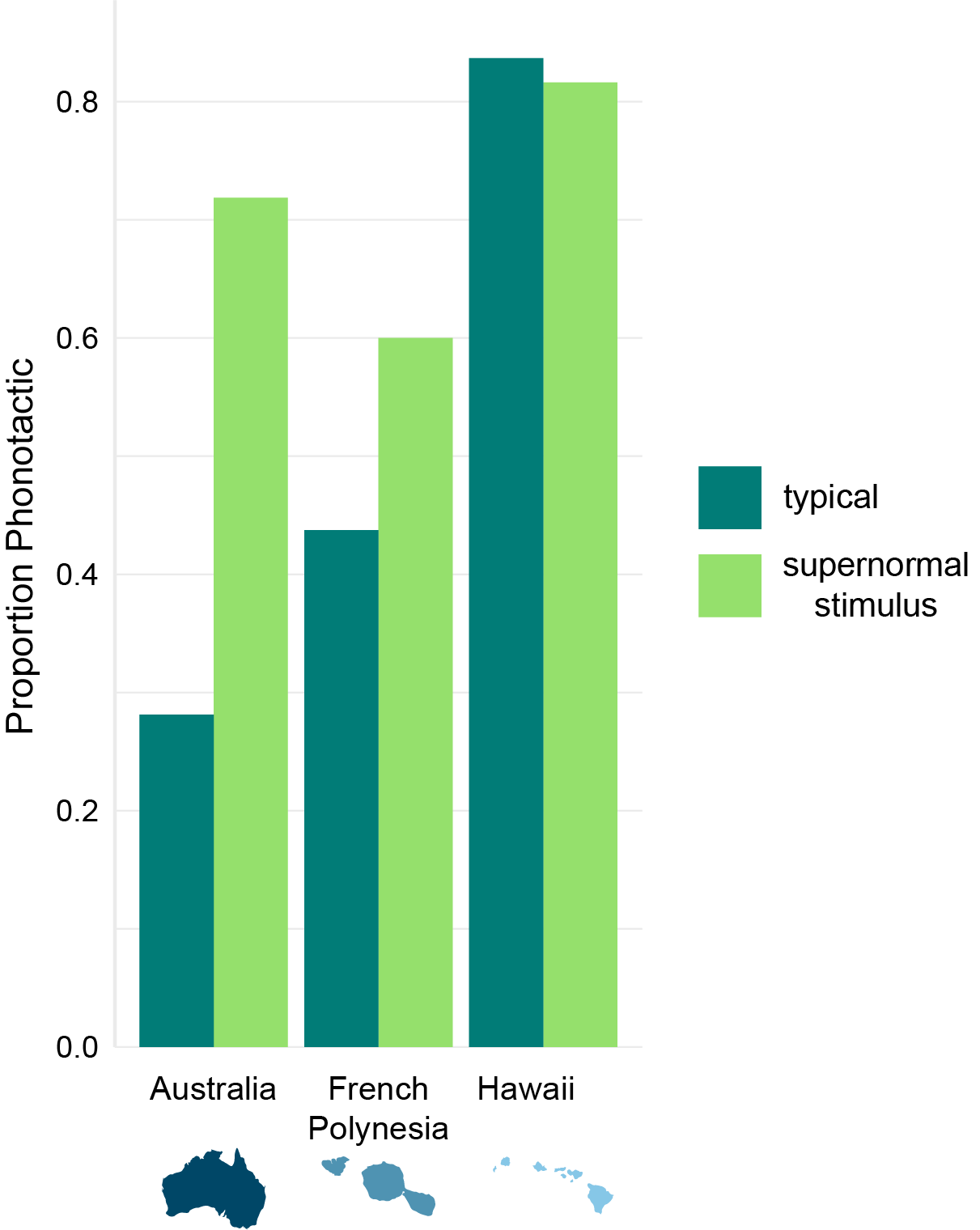
**

**Figure S2.** This shows field phonotaxis data in response to typical song and the supernormal stimulus in all regions. Females responded positively to the supernormal stimulus in all regions indicating that they were indeed reproductively mature.


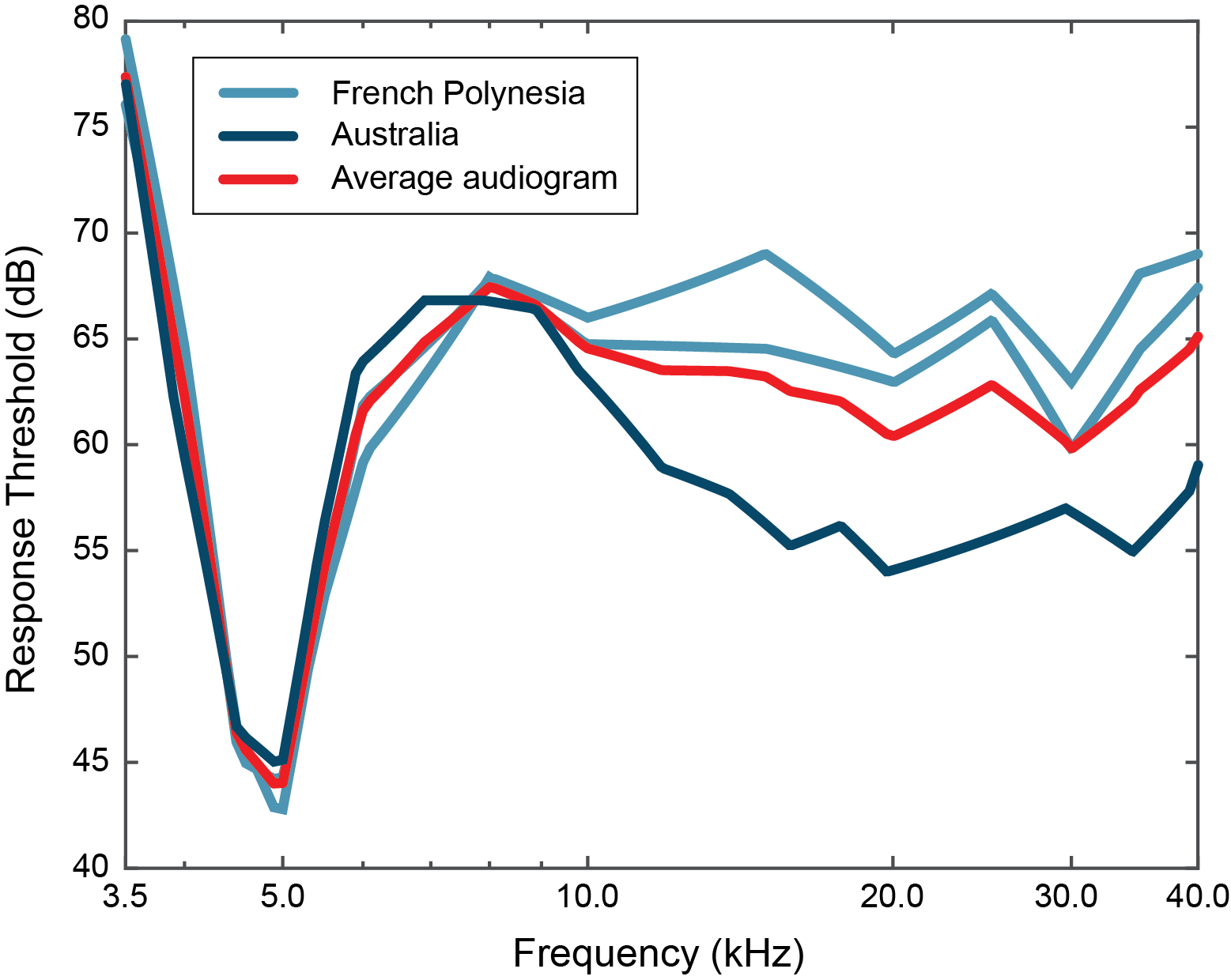


**Figure S3.** Neural audiograms of ON1 (omega neuron 1), extracted and replotted from Atkins & Pollack (1986) and Fullard et al. (2010). Data from Atkins & Pollack (1986), shown in dark blue, are from Australian crickets; data from Fullard et al. (2010), shown in light blue, are from Polynesian crickets. The average of all these audiograms are shown in red.


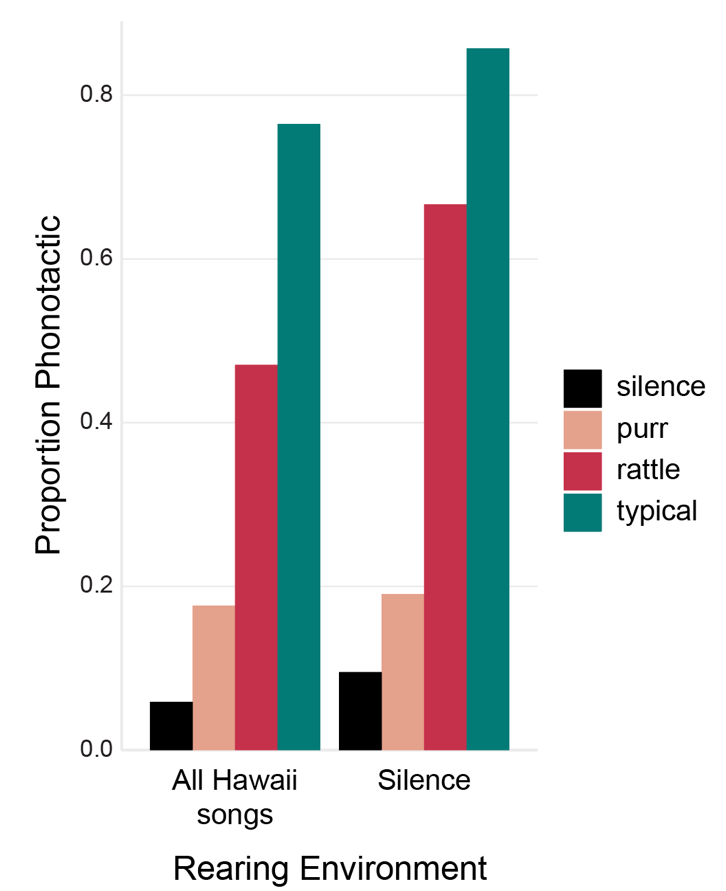


**Figure S4.** Phonotaxis data for Wailua showing the proportion of females that were positively phonotactic to silence (black), purring (light salmon), rattling (deep pink), and typical (teal) songs between populations reared in all the songs present in Hawaii (n=17) vs silence (n=21). All phonotaxis trials were conducted using the same exact methods. GLMM: song p= 0.002, rearing environment p= 0.896, song*rearing environment p=0.905.

Supplementary Methods:

*Generating a supernormal stimulus*

In the field, we were surprised to find that Australian females exhibited relatively low levels of phonotactic behavior towards the typical *T. oceanicus* calling song loop. To ensure this reflected strong preferences/discrimination and not females who weren’t yet phonotactic, we developed a supernormal stimulus while in the field by manipulating the typical loop using Audacity (version 2.3.1, The Audacity Team) to enhance the characteristics that are known to be preferred by females (Simmons 2001; Simmons 2004). We shortened the intercall duration and interpulse intervals by manually trimming the audio file, increased the proportion long chirp by trimming the short chirp section (therefore changing the long chirp:short chirp ratio), increased the amplitude using the amplify function, and increased the amplitude at the dominant frequency of 5 kHz using the Graphic EQ function. We played this supernormal stimulus to 161 females in all 6 locations, finding that 60.9% of females responded positively to the stimulus and confirming that the phonotaxis patterns observed reflected preference and not maturity.

References

Audacity (2025). Audacity (Version 2.3.1). https://[www.audacityteam.org/](http://www.audacityteam.org/)

Simmons, L. W., Zuk, M., & Rotenberry, J. T. (2001). Geographic variation in female preference functions and male songs of the field cricket *Teleogryllus oceanicus*. *Evolution*, 55, 1386-1394.

Simmons, L. W. (2004). Genotypic variation in calling song and female preferences of the field cricket *Teleogryllus oceanicus*. *Animal Behaviour*, 68, 313-322.
